# Plasma levels of Th17‐associated cytokines and selenium status in autoimmune thyroid diseases

**DOI:** 10.1002/iid3.433

**Published:** 2021-05-04

**Authors:** Tatjana Zake, Ieva Kalere, Sabine Upmale‐Engela, Simons Svirskis, Gita Gersone, Andrejs Skesters, Valerija Groma, Ilze Konrade

**Affiliations:** ^1^ Department of Internal Medicine Riga Stradins University Riga Latvia; ^2^ Institute of Microbiology and Virology Riga Stradins University Riga Latvia; ^3^ Department of Human Physiology and Biochemistry Riga Stradins University Riga Latvia; ^4^ Laboratory of Biochemistry Riga Stradins University Riga Latvia; ^5^ Institute of Anatomy and Anthropology Riga Stradins University Riga Latvia

**Keywords:** autoimmune thyroid disease, selenium, T helper 17 cells

## Abstract

**Introduction:**

The contribution of Th17 cytokines to autoimmune thyroid disease (AITD) is generally accepted. However, the roles of Th17 cells in the initiation and progression of Hashimoto's thyroiditis (HT) and Graves' disease (GD) remain unclear. Selenium deficiency, along with genetic predisposition and environmental factors, may have a role in thyroid autoimmunity.

**Aim:**

We aimed to assess (1) the Th17 immune response by measuring plasma levels of Th17‐ and Treg‐associated cytokines and (2) the selenium status in treatment‐naïve Latvian patients with newly diagnosed GD or HT.

**Methods:**

Eleven GD patients, 41 HT patients, and 26 healthy subjects were recruited for this study. Plasma levels of IL‐17a, IL‐22, IL‐23, IL‐6, and IL‐10 were detected by xMAP technology, while selenium was detected fluorometrically.

**Results and Conclusions:**

No significant differences in IL‐17a, IL‐22, IL‐23, IL‐6, or IL‐10 levels were found among the HT patients, GD patients, and controls. In the HT patients, IL‐17a levels were positively correlated with IL‐22, IL‐23, IL‐6, and IL‐10, while IL‐22 was correlated with IL‐6, IL‐23, and IL‐10. In the GD patients, IL‐17a levels were positively correlated with IL‐22, IL‐23, and IL‐10; IL‐22 was positively correlated with IL‐23, IL‐6, and IL‐10; FT3 was positively correlated with IL‐17a, IL‐23, and IL‐10; and FT4 was positively correlated with IL‐17a and IL‐10 levels. Plasma selenium levels were negatively correlated with antithyroid peroxidase antibody titers in the HT patients. Although no difference in selenium levels was observed between the AITD patients and controls, the selenium status of the Latvian patients with GD or HT was at a suboptimal level.

## Graphical abstract

Th17 cytokines were positively correlated with the severity of hyperthyroidism, suggesting their possible role in Graves' disease pathogenesis. Plasma selenium levels were negatively correlated with antithyroid peroxidase antibody titers in the Hashimoto's thyroiditis patients

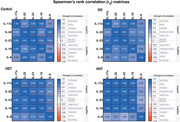

## 1 INTRODUCTION

Autoimmune thyroid disease (AITD), predominantly presenting as Graves' disease (GD) or Hashimoto's thyroiditis (HT), is the most frequent autoimmune disease with increasing prevalence and incidence in recent decades. In both autoimmune disorders, the complex interactions among various environmental and endogenous factors trigger thyroid autoimmunity in predisposed individuals, leading to enhanced thyroid autoantigen presentation and impairment of immune tolerance.^1^ In HT development, enhanced secretion of interferon (IFN)‐γ, interleukin (IL)‐1β, IL‐2, and tumor necrosis factor (TNF)‐α paralleled by thyroid cell apoptosis occurs due to the impaired T helper (Th)‐1‐based immune response, whereas in GD, upregulated production of IL‐4, IL‐5, IL‐6, and IL‐13, resulting from the Th2‐driven humoural response, causes the secretion of autoantibodies recognizing thyroid‐stimulating hormone receptor (anti‐TSHR) and hypertrophic changes.^2^

The understanding of the Th1 and Th2 cell dichotomy, which is essential for autoimmune disorders, shifted with the discovery of Th17 lymphocytes. Supporting evidence suggesting the contribution of Th17 cells and pro‐inflammatory IL‐17 to AITD, particularly HT, has been produced in the last decade.^3^, ^4^, ^5^, ^6^ It has been proposed that AITD results from Th1/Th2 dichotomy and Th17/T regulatory (Treg) cell imbalances.^7^ Th17 lymphocytes mainly secrete IL‐17 and IL‐22. IL‐17 is a critical pro‐inflammatory cytokine mediating chronic and autoimmune inflammation and neutrophil recruitment. Acting on immune and nonimmune cells such as epithelial cells, endothelial cells, fibroblasts, and osteoclasts, IL‐17 promotes the expression of various cytokines (IL‐1β, TNF‐α, IL‐6, IL‐8, granulocyte macrophage colony‐stimulating factor [GM‐CSF], and granulocyte colony‐stimulating factor), chemokines (CXCL1, CXCL5, and CCL2), and metalloproteinases by cells.^8^ The initial development of Th17 cells from naïve Th cells is induced by transforming growth factor (TGF)‐β in the presence of IL‐6 and IL‐21, while IL‐23 and IL‐1β are vital for both the complete maturation and stabilization of the Th17 phenotype.^9^, ^10^ To date, the association between selenium and Th17‐related cytokines has been insufficiently explored, mostly in experimental models.

Currently, a role for selenium deficiency in the development of AITD is suggested along with genetic predisposition and environmental triggers. Selenium deficiency can impair the differentiation of CD4+ T cells, leading to cellular and humoural response dysfunction. Selenium leads to changes in the levels of IL‐6, TGF‐β, and IL‐23 and may further contribute to impaired differentiation of Th17 cells.^11^ Very little information is currently available on the selenium content in soils in Latvia or the selenium status in AITD.

In this study, we aimed to assess (1) the Th17 immune response in AITD by measuring the plasma concentrations of Th17‐ and Treg‐associated cytokines and (2) the selenium status in Latvian patients with newly diagnosed, treatment‐naïve GD or HT to determine whether the selenium level is reduced in AITD patients compared to healthy subjects without autoimmune disorders.

## 2 MATERIALS AND METHODS

### 2.1 Patients

Eleven adult patients with untreated newly diagnosed hyperthyroid GD and 41 patients with untreated new‐onset HT were recruited into this prospective study between January and November 2020 at the Department of Endocrinology, Riga East University Hospital, Latvia. Thirty‐three out of 41 HT patients were euthyroid (median TSH, 1.90 [1.00–2.62] μIU/ml), and 8 out of the 41 patients had mild subclinical hypothyroidism (median TSH, 5.90 [5.41–7.35] μIU/ml). The median age was 36 (27–50.5) years for the HT patients, 41 (29–62) years for the GD patients, and 30 (27–42.25) years for the control group (*p* = .139). The diagnosis of AITD was confirmed by evaluating the clinical manifestations of GD and HT, complemented by biochemical thyroid tests and ultrasound imaging. The control group consisted of 26 age‐ and sex‐matched healthy subjects without any autoimmune disease and with normal thyroid function, who were negative for anti‐thyroid peroxidase autoantibodies (anti‐TPO), anti‐thyroglobulin autoantibodies (anti‐Tg), antinuclear antibodies (ANAs), and anti‐tissue‐transglutaminase IgA (tTG‐IgA) autoantibodies.

The exclusion criteria for the study included (1) pregnancy; (2) presence of malignancy, active infection, chronic inflammatory, or other autoimmune disease; (3) use of selenium‐containing commercial supplements; (4) vegetarian or vegan diet; (5) use of medications affecting thyroid and immune function (amiodarone, corticosteroids, nonsteroidal anti‐inflammatory drugs, antidepressants, or anticonvulsants); and (6) significant renal or hepatic impairment.

The study protocol was conducted according to the Declaration of Helsinki and approved by the Riga East Hospital Research Committee and the Central Medical Ethics Committee, Latvia (Decision No. 01‐29.1/5033). All patients and volunteers provided written informed consent before enrollment and blood sample collection.

### 2.2 Blood collection

Peripheral blood samples were collected from all 86 patients and volunteers after overnight fasting early in the morning. Thereafter, the samples were centrifuged at 1500 rpm for 10 min and stored at −80°C until analysis. Venous blood samples were collected from hyperthyroid and hypothyroid patients before therapy with methimazole or levothyroxine, respectively.

### 2.3 Thyroid function and antibody tests

The levels of serum‐free thyroxine (FT4), free triiodothyronine (FT3), and thyroid‐stimulating hormone (TSH), as well as anti‐TPO and anti‐Tg were measured by chemiluminescence immunoassay (Siemens) performed on an Advia Centaur XP (Siemens) analyzer. The normal values were as follows: FT4, 0.7–1.48 ng/dl; FT3, 0.2–0.44 ng/dl; and TSH, 0.35–4.94 μIU/ml; anti‐TPO, 0‐5.61 IU/ml; anti‐Tg, 0‐40 U/ml. The levels of anti‐TSHR, ANAs, and tTG‐IgA autoantibodies were measured by ELISA (Pharmacia Diagnostics Freiburg) according to the manufacturer's instructions (reference range: anti‐TSHR, 0–1.58 IU/L; ANA, reference range would be “negative”; tTG‐IgA, 0–10.0 U/ml).

### 2.4 Cytokines detection

Cytokine patterns analyzed in the current study included the following groups: Th17‐related cytokines (IL‐17a and IL‐22), Th17‐promoting cytokines (IL‐23 and IL‐6), and a Treg‐associated cytokine (IL‐10). EDTA plasma immunological markers were detected by xMAP technology (Magpix system; Luminex Corporation). All tests were performed in accordance with the manufacturer's instructions (Cat#: HTH17MAG‐14K; Kit Lot#: 3323752; Milliplex). The limit of detection for each cytokine was as follows: IL‐17a, 0.009 pg/ml; IL‐22, 0.021 ng/ml; IL‐23, 0.098 ng/ml; IL‐6, 1.7 pg/ml; and IL‐10, 0.3 pg/ml.

### 2.5 Selenium assay

The plasma selenium concentration was determined fluorometrically by using the fluorescence spectrophotometer “Cary Eclipse” (Varian, Inc.).^12^ Inter‐laboratory quality control was conducted by employing two standards—a selenium AAS solution (Cat# 24, 792‐8; Sigma‐Aldrich) and Seronorm TE Serum Level I (Cat# 201 405; Sero AS)—for the Seronorm™ Trace Elements‐Controls Programme. External quality assessment services were provided by Labquality Oy (Finland). The lower detection limit for selenium was 4 µg/L.

### 2.6 Statistical analysis

The normal distribution of data was confirmed by D'Agostino and Pearson, Anderson–Darling, and Shapiro–Wilk normality tests. Homogeneity of variances was tested using Brown–Forsythe and Bartlett's tests. As dispersion did not correspond to a normal distribution in most cases, data were analyzed by the nonparametric Kruska–Wallis *H* test followed by the two‐stage step‐up method of Benjamini, Krieger, and Yekutieli as a post hoc procedure, and the results are displayed as the median and interquartile range. Depending on the data distribution, both parametric Pearson's analysis and nonparametric Spearman's correlation analysis were performed to determine the relationships of studied parameters. *p* < .05 was considered statistically significant for all statistical tests. All graphical images and statistical analyses were performed using GraphPad Prism 9.0 for MacOS software (GraphPad Software).

## 3 RESULTS

**Figure 1:**
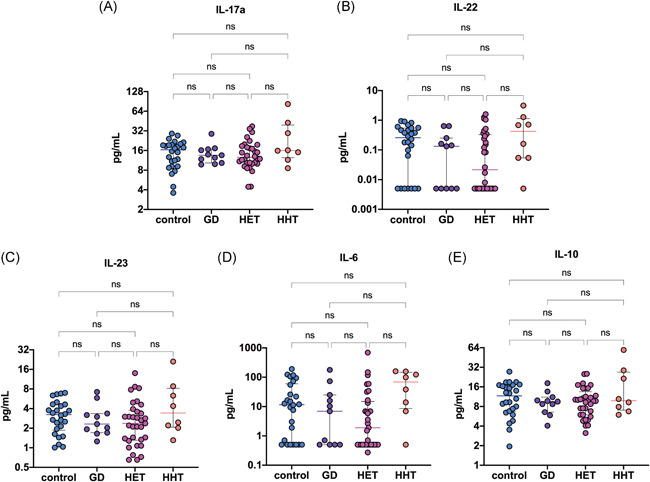
Comparisons of plasma IL‐17a, IL‐22, IL‐23, IL‐6, and IL‐10 levels among patients with HET (*n* = 33), HHT (*n* = 8), or GD (*n* = 11) and healthy subjects (*n* = 26). The dot plots represent the quantified data for (A) IL‐17a, (B) IL‐22, (C) IL‐23, (D) IL‐6, and (E) IL‐10 plasma levels. (A–E) Each dot represents a single data point. ns, not significant; GD, Graves' disease; HET, Hashimoto's thyroiditis, euthyroidism; HHT, Hashimoto's thyroiditis, hypothyroidism

The demographic and biochemical characteristics of the participants are presented in Table 1. HT patients were stratified into two groups according to their serum TSH level: euthyroid (0.354 ≤ TSH ≤ 4.94 μIU/ml; *n* = 33) and hypothyroid (4.94 < TSH < 10 μIU/ml; *n* = 8) patients. No significant differences in IL‐17a, IL‐22, IL‐23, IL‐6, or IL‐10 levels were found among the patients with HT, patients with GD and controls (all *p* > .05) (Figure 1). Interleukin levels also did not differ between the euthyroid and hypothyroid HT patients (all *p* > .05). The plasma levels of cytokines are summarized in Table 2.

**Table 1:** Demographic and biochemical characteristics of the participants

	HT patients (*n* = 41)	GD patients (*n* = 11)	Controls (*n* = 26)	*p*
Sex (female/male)	39/2	9/2	21/5	.152
Age (years)	36 (27–50.5)	41 (29–62)	30 (27–42.25)	.139
TSH (μIU/ml)	2.16 (1.42–3.77)	0.0003 (0.0000–0.0004)	1.11 (0.96–1.67)	<.001
FT4 (ng/dl)	0.91 (0.85–0.98)	1.97 (1.76–2.55)	0.94 (0.91–1.07)	<.001
FT3 (ng/dl)	0.32 (0.29–0.35)	1.21 (1.04–2.47)	0.32 (0.30–0.35)	<.001
Anti‐TPO (IU/ml)	202.99 (75.80–421.40)	121.04 (3.74–929.28)	0.46 (0.25–0.78)	<.001
Anti‐Tg (U/ml)	0.00 (0.00–51.02)	5.96 (0.00–43.46)	<20	
Anti‐TSHR (IU/L)	<1.58	9.18 (4.05–19.68)	<1.58	

*Note*: Data are presented as the median (interquartile range). The Kruskal–Wallis test was used to calculate the *p* value for comparisons among three groups for non‐normally distributed data.

Abbreviations: FT3, triiodothyronine; FT4, thyroxine; GD, Graves' disease; HT, Hashimoto's thyroiditis; Tg, thyroglobulin; TPO, thyroid peroxidase; TSH, thyroid‐stimulating hormone; TSHR, thyroid‐stimulating hormone receptor.

*p* < .05: statistically significant.

**Table 2:** Plasma levels of interleukins in the study groups

	HT patients (*n* = 41)		GD patients (*n* = 11)	Controls (*n* = 26)	*p*
	Euthyroid HT (n = 33)	Hypothyroid HT (*n* = 8)			
IL‐17a (pg/ml)	14.83 (10.37–19.75)		13.53 (10.33–16.72)	16.43 (9.14–19.75)	.906
	12.95 (10.18–19.18)	15.99 (12.38–39.37)			.606
IL‐22 (ng/ml)	0.08 (0.00–0.47)		0.13 (0.00–0.25)	0.25 (0.00–0.54)	.393
	0.026 (0.000–0.351)	0.421 (0.054–1.138)			.180
IL‐23 (ng/ml)	2.45 (1.35–4.49)		2.31 (1.70–3.36)	3.23 (1.86–4.64)	.653
	2.37 (1.30–3.76)	3.42 (2.06–8.18)			.408
IL‐6 (pg/ml)	5.88 (0.00–36.24)		6.92 (0.00–24.90)	11.61 (0.00–59.50)	.682
	1.90 (0.00–14.93)	68.62 (8.67–148.25)			.059
IL‐10 (pg/ml)	9.70 (6.35–15.21)		9.50 (6.54–11.11)	11.62 (6.70–17.04)	.658
	9.70 (5.96–13.47)	9.83 (7.03–26.90)			.585

*Note*: Data are presented as the median (interquartile range). The Kruskal–Wallis test was used to calculate the *p* value for comparisons among three groups for non‐normally distributed data.

Abbreviations: GD, Graves' disease; HT, Hashimoto's thyroiditis.

*p* < .05: statistically significant.

**Figure 2:**
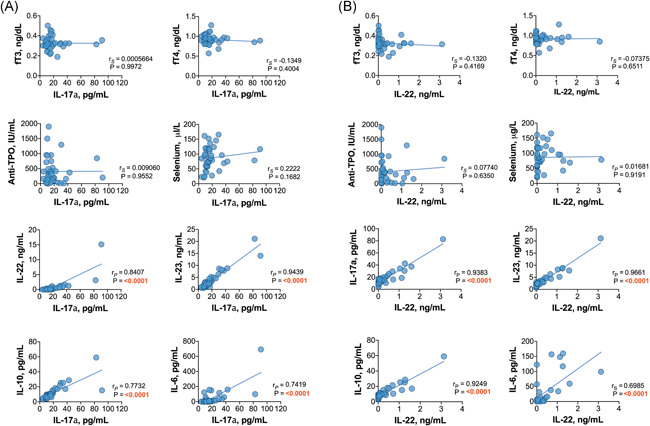
Relationships between (A) IL‐17a or (B) IL‐22 and the studied biomarkers in the Hashimoto's thyroiditis patient group

**Figure 3:**
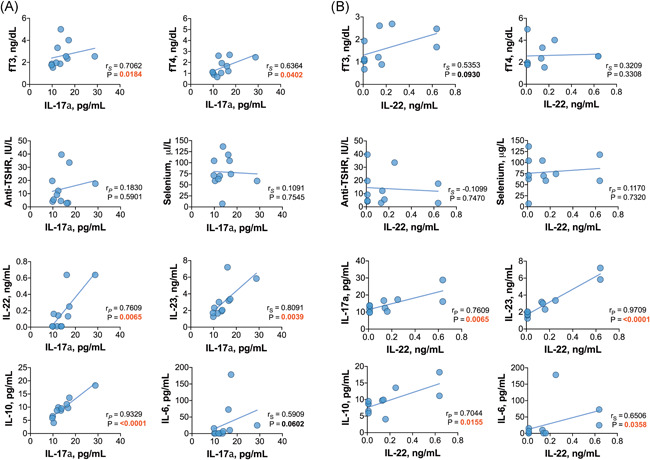
Relationships between (A) IL‐17a or (B) IL‐22 and the studied biomarkers in the Graves' disease patient group

**Figure 4:**
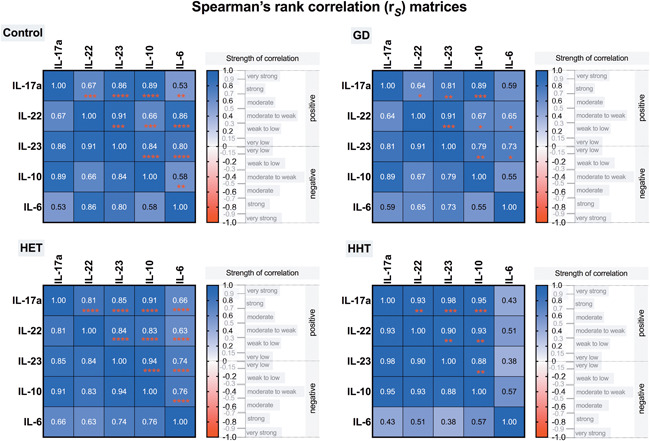
Variance–covariance matrices with Spearman's rank correlation coefficients (values in small squares) representing the association level between studied cytokines in patient groups. GD, Graves' disease; HET, Hashimoto's thyroiditis, euthyroidism; HHT, Hashimoto's thyroiditis, hypothyroidism. *p < .05; **p < .01; ***p < .001; ****p < .0001

When the relationships among Th17‐related cytokines were investigated, we found that in HT patients, the plasma levels of IL‐17a were positively correlated with the levels of IL‐22, IL‐23, IL‐6, and IL‐10 (Figure 2A); additionally, IL‐22 was correlated with IL‐6, IL‐23, and IL‐10 (Figure 2B). Similarly, in GD patients, positive associations between IL‐17a and the levels of IL‐22, IL‐23, and IL‐10 were found; however, IL‐17a was not correlated with IL‐6 (Figure 3A). There were positive correlations between the levels of IL‐22 and those of IL‐23, IL‐6, and IL‐10 in patients with GD as well (Figure 3B). Positive relationships between different interleukins were revealed in the control group and both euthyroid HT patients and hypothyroid HT patients. Variance–covariance matrices with Spearman's rank correlation coefficients and *p* values representing the association level between studied cytokines in the patient groups are given in Figure 4.

**Figure 5:**
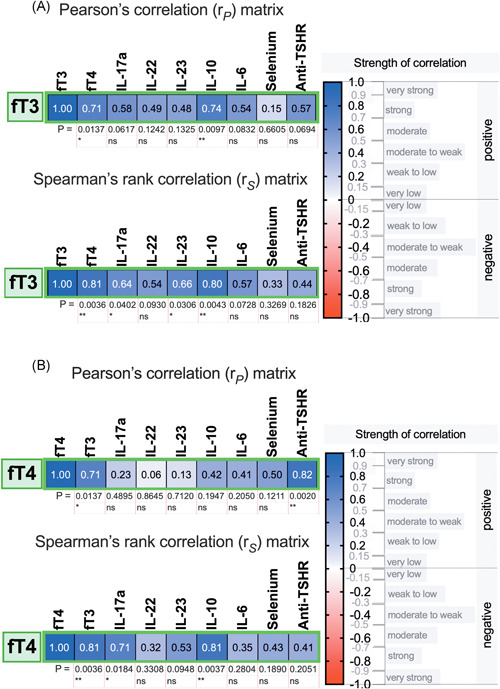
Associations of (A) fT3 or (B) fT4 with the studied biomarkers in the GD patient group. ns, not significant; *p < .05; **p < .01. fT3, triiodothyronine; fT4, thyroxine; GD, Graves' disease; TSHR, thyroid‐stimulating hormone receptor

Moreover, no significant correlations were found between the levels of interleukins and thyroid autoantibodies, TSH, FT3 or FT4 in HT patients (all *p* > .05). However, in newly diagnosed GD patients, the levels of FT3 were positively correlated with IL‐17, IL‐23, and IL‐10 (Figure 5A), while FT4 was positively correlated with IL‐17 and IL‐10 levels (Figure 5B). In addition, anti‐TSHR autoantibody titers were not correlated with the levels of interleukins in the GD group (all *p* > .05).

**Figure 6:**
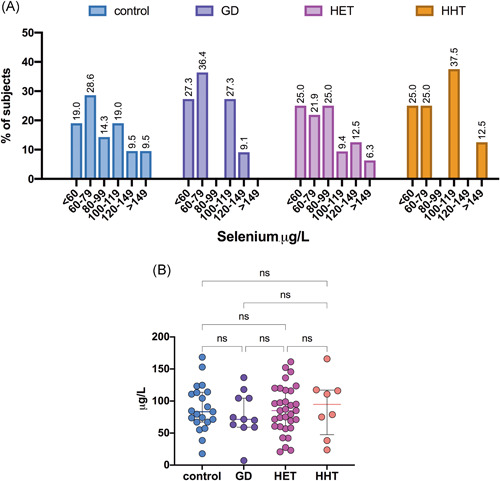
Histograms showing the frequency distribution of plasma selenium (A) and comparison of selenium levels (B) in patient groups. (B) Each dot represents a single data point. GD, Graves' disease; HET, Hashimoto's thyroiditis, euthyroidism; HHT, Hashimoto's thyroiditis, hypothyroidism; ns, not significant

**Figure 7:**
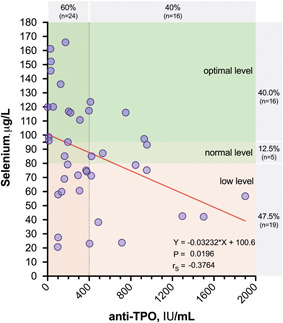
Correlation between selenium and anti‐TPO autoantibody levels in patients with HT. Plasma selenium level below 80 μg/L was defined as low, 80–95 μg/L as normal, and the level above 95 μg/L as optimal. HT, Hashimoto's thyroiditis; TPO, thyroid peroxidase

The median plasma selenium levels were 85.03 (60.09–116.60) µg/L for HT patients, 71.33 (59.05–104.42) µg/L for GD patients, and 80.35 (67.15–113.67) µg/L for control participants (Table 3). The plasma selenium distribution in the patient groups is presented in Figure 6A. The levels of selenium did not differ significantly among the studied groups (Figure 6B). Similarly, no differences in selenium levels were observed between euthyroid HT patients and HT patients with subclinical hypothyroidism (Table 3). Interestingly, we found that plasma selenium levels showed a negative correlation with anti‐TPO autoantibody titers in patients with HT (*r* = −.376; *p* = .02) (Figure 7). In addition, significantly lower selenium levels were observed in HT patients with higher anti‐TPO levels (≥400 IU/ml) than in those with lower autoantibody titers (<400 IU/ml) (72.75 [52.10–88.43] vs. 89.04 [65.53–117.51] µg/L, respectively; *p* = .05).

**Table 3:** Plasma levels of selenium in the study groups

	HT patients (*n* = 40)	
	Euthyroid HT (n = 32)	Hypothyroid HT (n = 8)	GD patients (*n* = 11)	Controls (*n* = 21)	*p*
Se (µg/L)	85.03 (60.09–116.60)		71.33 (59.05–104.42)	80.35 (67.15–113.67)	.763
	85.03 (60.09–116.56)	94.78 (47.46–117.02)			.886

*Note*: Data are presented as the median (interquartile range).

When the associations between plasma selenium and cytokines were analyzed, no significant correlations were found between selenium levels and the plasma levels of IL‐17a, IL‐22, IL‐23, IL‐6, and IL‐10 for all groups (all *p* > .05). The levels of selenium failed to correlate with other characteristics, such as age, titers of anti‐Tg and anti‐TSHR, TSH, FT3, and FT4 (all *p* > .05).

## 4 DISCUSSION

Cytokines, along with T cell receptors, transcription factors, and the intestinal microbiota, are important factors regulating the Th17/Treg cell balance. Th17 cells have been implicated in the development of autoimmunity, whereas Treg cells producing IL‐10, IL‐35, and TGF‐β maintain self‐tolerance and protect against thyroid autoimmunity. Moreover, IL‐10 is a principal anti‐inflammatory cytokine that has been shown to inhibit Th17 cell‐mediated inflammation.^13^ Furthermore, current evidence suggests the Th17 lineage is plastic and heterogeneous.^7^ Another pro‐inflammatory cytokine contributing to thyroid autoimmunity is IL‐23. Produced by antigen‐presenting cells such as macrophages and dendritic cells, IL‐23 is crucial for the differentiation of pathogenic Th17 lymphocytes.^14^ Furthermore, it has been found to stimulate the survival of highly pathogenic Th17 cells.

Only limited data are available for serum IL‐23 and IL‐22 levels in AITD patients. Shi et al.^15^ demonstrated upregulated messenger RNA (mRNA) expression of IL‐17 and the Th17‐related transcription factor RORγt in peripheral blood mononuclear cells along with elevated plasma levels of IL‐17 and IL‐23 in newly diagnosed HT patients. More recently, Konca Degertekin et al.^3^ demonstrated lower serum levels of IL‐17 and IL‐23 in hypothyroid HT patients than in euthyroid patients, thus suggesting that hypothyroidism has an inhibitory impact on Th17 cytokine responses. However, no differences in IL‐17 or IL‐23 levels were found between hypothyroid HT patients and healthy subjects. Elevated levels of serum IL‐23 were found in untreated euthyroid HT patients compared with controls, reflecting the early course of thyroiditis, in a study conducted by Ruggeri et al.^16^ Jia et al.^17^ observed increased serum levels of IL‐23 in GD patients. In contrast, Figueroa‐Vega et al.^4^ did not find any significant differences in IL‐23 levels between GD patients and healthy subjects. Previously, we demonstrated thyrocyte‐related IL‐23 overexpression in HT patients, while no difference was found between GD and colloid goiter patients, suggesting that IL‐23 may be less implicated in GD pathogenesis than in HT pathogenesis.^18^

IL‐22 produced by Th17 cells can also be secreted by another novel subset of Th cells—CCR^+^IL‐17^−^IL‐22^+^ (Th22) cells. We failed to demonstrate any differences in IL‐22 levels among the three groups of subjects enrolled in this study. However, Ruggeri et al.^19^ found significantly increased serum levels of IL‐22 in untreated euthyroid HT patients compared with patients with nodular goiter or healthy subjects. In another study, drug‐naïve HT patients suffering from overt or subclinical hypothyroidism exhibited elevated serum levels of IL‐17, while no differences were found in IL‐22 or IL‐23 levels between the HT patients and healthy individuals. Song et al.^20^ reported that peripheral blood Th22 cell levels, serum IL‐22 levels, and IL‐22 mRNA expression were elevated in GD patients compared with controls, suggesting that IL‐22 may participate in the initiation of GD. Similar to the results confirmed in the present study, significant differences in the Th22 profile were not found in HT patients.

In this study, positive correlations between the levels of IL‐22, IL‐17a, IL‐23, and thyroid hormones were found in newly diagnosed GD patients. However, no correlations between the levels of interleukins and thyroid‐specific autoantibodies were found. These results suggest that Th17 cytokines may have a stimulatory effect on the severity of hyperthyroidism independent of autoantibody levels. No such correlation was found in the HT group as a vast majority of the recruited HT patients presented as euthyroid. Due to the limited availability of treatment‐naïve patients, only a small number of GD patients was recruited. Therefore, the results of GD group analyses must be concluded and interpreted with caution because of the small sample size in this group.

Among the nongenetic factors involved in the pathogenesis of thyroid autoimmunity, high iodine intake, and a low selenium status appear to be the most important. Previous studies have suggested the immunomodulatory and protective role of selenium and selenoproteins. It has been suggested that selenium deficiency presents with inhibition of selenoproteins and altered secretion of IL‐10, IL‐12p40, and IFN‐γ, leading to Th1/Th2 imbalance by shifting the balance toward Th1 responses.^21^ Additionally, glutathione peroxidase (GPx)1‐deficient Th lymphocytes demonstrate a shift toward Th1 cells and attenuate the differentiation of Th cells into the Th17 lineage.^22^ In another study, selenium supplementation exerted protective effects against oxidative stress and peroxide‐induced cell damage in human thyrocytes and fibroblasts.^23^ However, more data from clinical studies are needed for understanding the relationship between selenium supplementation, immune response, and antioxidant effects. In the current study, we did not find any correlations between the plasma levels of selenium and interleukins within the patient groups, indicating that adequate selenium intake does not skew the differentiation of CD4+ T cells toward the Th1 or Th2 lineage and that Th1/Th2 differentiation is largely determined by the pattern of secreted cytokines, transcription factors, and other environmental signals.

In a population‐based Danish study, Pedersen et al.^24^ explored serum selenium levels in patients with newly diagnosed AITD. The authors found significantly lower selenium levels in patients with GD than in controls; however, no difference in serum selenium levels between HT patients and healthy subjects was observed. In 2014, a multicentre, cross‐sectional study was conducted in Austria, Greece, Romania, and Italy to assess the selenium status of euthyroid patients with thyroid autoimmunity and those with non‐autoimmune thyroid diseases.^25^ The study confirmed the presence of a strong positive correlation between the plasma levels of selenium and selenoprotein P (SePP), indicating a less‐than‐optimal selenium status in these European countries. Additionally, significantly lower plasma selenium levels were found in patients with GD or HT than in those with non‐autoimmune thyroid disease, suggesting that selenium supplementation might be beneficial in the case of thyroid autoimmunity. Federige et al.^26^ also explored serum selenium and SePP levels in AITD patients. Similar to the current study, no difference in serum selenium levels was found among HT patients, HT + LT4 patients, GD patients with or without GO, and healthy individuals, although SePP levels were lower in the GO and HT patients than in the control subjects.

Various plasma or whole‐blood selenium cut‐offs have been proposed by different authors to reflect selenium sufficiency. The activity of GPx3 in human serum is used as a marker of selenium sufficiency. It has been shown that the plasma selenium level needed to optimize platelet GPx activity is 100–115 μg/L, as proposed by Alfthan et al.,^27^ or 95 μg/L according to Thomson et al.,^28^ whereas a plasma selenium range of 88–142 µg/L is associated with the maximal SePP1 concentration.^29^ A selenium level below 80 μg/L is considered to indicate an inadequate selenium status.^30^, ^31^ The aforementioned cut‐offs suggest that 35% of the HT patients and 36.4% of the GD patients enrolled in the study had plasma selenium levels necessary for saturation of GPx, while 47.5% of the HT patients and 63.3% of the GD patients had a poor selenium status. The frequency of selenium deficiency in the control group was 47.6%.

## 5 CONCLUSIONS

Despite the recent data on the involvement of the IL‐17/IL‐23 axis in the development of thyroid autoimmunity available in the literature, we did not find any significant differences in the plasma levels of Th17‐associated cytokines between patients with AITD and control subjects. However, a positive correlation between Th17 ILs was detected in both HT patients and GD patients. Furthermore, Th17 cytokines were positively correlated with the severity of hyperthyroidism, suggesting their possible role in GD pathogenesis. A few limitations should be considered when interpreting our data. Although simultaneous assessment of various cytokines provided more information on the complex cytokine pattern that characterizes AITD, a small number of AITD patients may lead to a possible loss of statistical significance. Due to the lack of data regarding the levels of other Treg cytokines, it will be of interest to further pursue studies of Th17 and Treg cells as important players in AITD pathogenesis. Although no difference in selenium levels was observed between AITD patients and controls, the selenium status of Latvian patients with newly diagnosed GD or HT was at a suboptimal level. Interestingly, plasma selenium levels were negatively correlated with anti‐TPO autoantibody titers in HT patients. Moreover, HT patients with higher anti‐TPO levels had lower levels of selenium, suggesting that these patients might benefit from selenium supplementation.

## AUTHOR CONTRIBUTIONS

Ilze Konrade and Tatjana Zake designed the study protocol, Tatjana Zake, Sabine Upmale‐Engela, Ieva Kalere, Gita Gersone, Andrejs Skesters collected the data and performed patient selection, Gita Gersone and Andrejs Skesters performed laboratory work, Tatjana Zake, Ieva Kalere, Sabine Upmale‐Engela, Gita Gersone, Simons Svirskis, Andrejs Skesters analyzed and interpreted the data, Tatjana Zake, Ieva Kalere, Valerija Groma, Ilze Konrade, Simons Svirskis wrote and edited the manuscript, Ilze Konrade and Valerija Groma supervised the study. All authors read and approved the final manuscript.

## CONFLICT OF INTERESTS

The authors declare that there are no conflict of interests.

## Data Availability

The data that support the findings of this study are available from the corresponding author upon reasonable request.
